# Supplementary material for: Impact of COVID-19 on feto-maternal and neonatal health in Karachi, Pakistan, A retrospective cohort study
Source: PLOS Glob Public Health. 2023 Aug 11;3(8):e0002139. doi: 10.1371/journal.pgph.0002139 (PMC10420353; doi:10.1371/journal.pgph.0002139)
Supplement: Attachment — Submitted filename: Response to Reviewers.docx [file pgph.0002139.s002.docx]

| **S.no** | **Additional Reviewer Comment** | **Responses** |
| --- | --- | --- |
| 1 | Please provide separate figure files in .tif or .eps format. | Figure file provided separately. |
| 2 | In the online submission form, you indicated that "Data is a intellectual property of Aga Khan university Hospital and is available on request from the corresponding author.". All PLOS journals now require all data underlying the findings described in their manuscript to be freely available to other researchers, either 1. In a public repository, 2. Within the manuscript itself, or 3. Uploaded as supplementary information. | Data file uploaded as supplementary information. |
| 3 | Please add a list of abbreviations | List added. |
| 4 | Mothers enrolled and babies enrolled number is different. | Because One mother had delivered twin babies, the mother number and baby num are different. |
| 5 | Use one referencing style. The author’s names in the Discussion text is Harvard style whereas the rest of the paper uses Vancouver | Only one referencing style used. |
| 6 | Use 'COVID-19' term in standard format | Standard format used. |
| 7 | Limitation do not talk about confounders | Correction done. |
| 8 | Conclusion is interpretation of results, and does not furnish any recommendations or way forward. | Correction rewritten. |
| 9 | Acknowledgements says thanks to participants; but it was a retrospective study which means you used the hospital data only. | Correction done. |
