# Supplementary material for: Impact of COVID-19 on feto-maternal and neonatal health in Karachi, Pakistan, A retrospective cohort study
Source: PLOS Glob Public Health. 2023 Aug 11;3(8):e0002139. doi: 10.1371/journal.pgph.0002139 (PMC10420353; doi:10.1371/journal.pgph.0002139)
Supplement: Attachment — Submitted filename: Response to reviewer.docx [file pgph.0002139.s003.docx]

“Impact of COVID-19 on Feto-maternal and Neonatal Health in Karachi, Pakistan, A retrospective cohort study” (PGPH-D-22-01826R1)

**Response to Reviewer Recommendations:**

| **S. #** | **Reviewer 1 recommendations:** | **Investigator’ Responses:** |
| --- | --- | --- |
| 1. | The statistical methods are rather poor and symplistic. Do they have an hystorical cohort before the pandemic to perform a comparisons in order to produce the extent of risk increase due to COVID 19. Thsi may be due easily with bivariate logistic regression adjusting for a handful of major confounder if possible (maternal age, parity,morbidities etc). | The suggestion has been duly noted and the study design has been changed to a case-control to allow for a comparision between pregnant women with and without COVID-19 who delivered.  Relevant statistical tests have been run better provide an understanding of risks associated with COVID-19 in pregnant women. |
| 2. | Can they provide a risk related to severity of COVID 19 for each abnormal obstetric outcomes? | Table 2 provides a comparison of risk of maternal and neonatal outcomes in cases and controls. |
| 3. | The references list is poor and should be enriched and so the discussion. There are important missing references describing association of COVID 19 to gestational diabete mellisus in the discussion (ref 1) and the same can be stated for preeclampsia (2). These references should be used to enrich the discussion of the results in the context of what is already known. | Recommendations noted nad changes made |
| 4. | The risk of thrombosis after surgery is increased in covid 19 thi salso applies to obstetric ijnterventions (2). Do they have a measure of trhormotic complications? If not disclose this limitation and add the reference to the list along wioth a short note presenting the issue. (ref 3) | This is a very pertinent observation. While literature suggests that the risk of thrombosis increases in severe COVID and in pregnancy, literature however did not show an increased risk when compared to non-pregnant women with severe COVID. This study was an observational one and therefore beyond the scope to determine if any thrombolytic event occurred in the post partum period. None occurred for neither cases nor controls during the study time period. |
| 5. | The quality of figure 1 is so low that I cannot read easily the content. Please reupload it with higher quality/definition | The Figure has been uploaded again with imroved quality. |
| 6. | Which was the viral variant of concern in Pakistan in that period? What percentage of the population was vaccined in the beginning and end of the study? This information should be added in the discussion according to the available local national literature/data. | COVID vaccination started in March 2021 with health care workers receiving the first doses. This was followed by elderly and then healthy adults in by mid of 2021. Pregnant women weren’t vaccinated till last quarter of 2021 and there was much speculation surrounding it’s safety. We thereore think that the impact of vaccination may not be so visible on this population.  Khan S, Khan SH, Haider F, Malik J, Khan F, Khan I, Ullah I, Zia MA, Kousar S. The Burden of Omicron Variant in Pakistan: An Updated Review. COVID. 2022; 2(10):1460-1476. <https://doi.org/10.3390/covid2100105> (covid spikes)  Imran M, Khan S, Khan S, Uddin A, Khan MS, Ambade P. COVID-19 situation in Pakistan: A broad overview. Respirology. 2021 Sep;26(9):891-892. doi: 10.1111/resp.14093. Epub 2021 May 31. PMID: 34056791; PMCID: PMC8242540.  (vaccination and spikes) |
| 7. | The strenght of the study is that describes the clinical picture in Pakistan, and there are limited data for this country. | The comment is appreciated as it was the aim of the authors to share knowledge. |
| 8. | In the conclusions I would reccomend to remember that given the high extent of complications in pregnancies affected by CODID 19 vaccine should be reccomended to all pregnant women as well as protection with facial masks. | This suggestion has been elaborated on in the Conclusions section. |
| Reviewer 2 | | |
| 1. | The design is a Retrospective Chart Review (RCR), recommended to change it | As per recommendation the study design has now been changed to case control. |
| 2. | There are several limitations of RCR which need to be elaborated in the methods/limitation section The study only uses descriptive analysis - itself a limitation Referencing need to be corrected/some references seem missing - intext and references section at the end | Thank you for your recommendations. The study design is no longer RCR but a case control. The analysis now comprises Referencing has been has been updated. |
| 3. | The manuscript requires native English editor copy editing and proofing | The manuscript has been revised as advised. |

*Name of responder / PI* : Dr. Ali Faisal Saleem

*Department:* Pediatrics and Child Health

*Date:* 25th March 2023
