# Supplementary material for: Impact of COVID-19 on feto-maternal and neonatal health in Karachi, Pakistan, A retrospective cohort study
Source: PLOS Glob Public Health. 2023 Aug 11;3(8):e0002139. doi: 10.1371/journal.pgph.0002139 (PMC10420353; doi:10.1371/journal.pgph.0002139)
Supplement: Attachment — Submitted filename: Response to reviewer.docx [file pgph.0002139.s004.docx]

“Impact of COVID-19 on Feto-maternal and Neonatal Health in Karachi, Pakistan, A retrospective cohort study” (PGPH-D-22-01826R2)

**Response to Reviewer Recommendations:**

| **S. #** | **Reviewer 1 recommendations:** | **Investigator’ Responses:** |
| --- | --- | --- |
| 1. | Reviewer #1: The revision slightly improved the manuscript. However, I noted that some of my previous comments were not considered by the authors such as the discussion of the major evidences emerging from the Intercovid study (ref 1-2) on preeclampsia and gestational diabetes and the discussion on risk of thrombosis (ref 3). It is interesting to observe that GDM is not significant on their data and this should be discussed in light of other major data with different and significant results (ref 2) | The Intercovid study ref (1-2) and (ref 3) were reconsidered and the recommeded portions added in as part of the discussion. |
| 2. | Preeclampsia disappeared from the table comparing study groups. Why? This outcome should be added as it is an essential outcome in covid-19 cases (please see ref n 2). | Preeclampsia as a maternal adverse event was not significant on the the multivariate model and therefore was removed from the table. It has been added to the Table 2 again keeping in consideration your recommendation. It is however observed that the adjusted odds ratio for preeclampsia is insignificant. |
| 3. | Moreover, recently, after the first submission, another major evidence emerged showing a significantly improved outcome after COVID 19 vaccine. Women with complete or boosted vaccine doses had reduced risk for severe symptoms, complications, and death (ref 4) Please add in the conclusion a plea for vaccining pregnant women this is essential as a public health intervention! Consider also citing major reference 4. | As mentioned in the response to previous reviewer comments, it was beyond the scope of the study to determine the impact of vaccination on pregnant women. It is however, indisputable that vaccination against COVID-19 not only prevents serious outcomes in women but also in the infants born to them. The plea for vaccination pregnant women had been mentioned earlier, but further emphasis has been added in the conclusion as recommended. |
| 4. | The conclusion should be restructured: there are several neonatal outcomes which are worst in covid 19 positive as compared to covid 19 negative patients and this does not emerge in the conclusions. More so the study is underpowered to address some rare and severe outcome: maternal death, intensive care unit, severe pneumonia. | There are two important points highlighted in this comment. Evidentially the manner in which the data had initially been analyzed changed with the change in methodology, hence warranting the restructuring of the conclusion. This has been done.  The second point brought to light is the power of the study which is correct, and this has been added to the limitations section. |
| 5. | Univariable statistics is rather simplistic approach and I recommend multivariable regression model considering some covariate. | As recommended multivariate regression model has been applied. The results have been incorporated and the Tables amended as per need. |
| 6. | "Pre rupture of membrane (85.7%)" is not sound: premature rupture of membranes English language should be revised "NICU admission coincided with increased infection...." Unclear... rephrase please. | The spelling error has been corrected as advised and the article has been reviewed to correct grammatical errors especially the mentioned portion. |

*Name of responder / PI* : Dr. Ali Faisal Saleem

*Department:* Pediatrics and Child Health

*Date:* 25th May 2023
